# Confusion Reigns: An Analysis of Responses to U.S. Soccer Age Cut-Off Date Policy Change

**DOI:** 10.3389/fspor.2021.635195

**Published:** 2021-03-25

**Authors:** Kristy L. Smith, Sara Scarfone, Laura Chittle, Sean Horton, Jess C. Dixon

**Affiliations:** Department of Kinesiology, University of Windsor, Windsor, ON, Canada

**Keywords:** U.S. soccer, age cut-off, date change, policy change, organizational change, relative age, youth sport, stakeholder response

## Abstract

Relative age effects (RAEs) have been associated with the common practice of grouping athletes by chronological age. Development and selection advantages are often awarded to those who are born closer to, but following, the cut-off date employed by sport systems. In 2015, the U.S. Soccer Federation announced that it would be changing its birth-year registration cut-off date from August 1st to January 1st. This change was introduced to align the U.S. youth soccer calendar with international standards, and simultaneously provide clearer information on player birthdates to “lessen” RAEs. The magnitude of this policy change has led to considerable controversy, with members of the soccer community taking to social media and website blogs, as well as the U.S. Youth Soccer's website, to voice their opinions and general unhappiness with this decision. Thus, the purpose of this study was to provide a summary of online reactions to the policy change, with attention to the manner in which the U.S. Soccer Federation framed (i.e., the underlying rationale for the decision) and publicly communicated its decision to change the annual cut-off date. Qualitative content analysis was used to analyze data collected from 63 social media sites (websites, *n* = 43; forums, *n* = 16; blogs, *n* = 4). From the 3,851 pages of text derived from these sources, a total of 404 unique passages of text were identified within 262 stakeholder posts. Four categories emerged from the data: stakeholder discussion, outcomes identified by stakeholders, recommended courses of action, and communication regarding the policy change. In general, the actions of the U.S. Soccer Federation and related outcomes were negatively perceived by stakeholders at various levels of the sport. Resistance to the change may have been reduced through enhanced communication from the national level and opportunities for stakeholder input. While one objective of this policy change was to combat RAEs, previous research suggests this organizational change will only shift which group of athletes experience relative age (dis)advantages. There appears to be a disconnect between the academic literature and sport policy with respect to solutions for RAEs, which can lead to unintended consequences for various sport stakeholders.

## Introduction

Relative age effects (RAEs) describe disparities in developmental outcomes that have been associated with the common practice of grouping children and youth by chronological age (Barnsley et al., 1985; Wattie et al., 2008). The use of these age groupings is well-intended, with the aim to provide developmentally appropriate instruction and competition. Yet, evidence to the contrary has been compiled in various domains such as organized sport participation (e.g., Cobley et al., 2009; Smith et al., 2018), academic performance (e.g., Bedard and Dhuey, 2006), high school leadership activities (e.g., Dhuey and Lipscomb, 2008), and even chess (e.g., Helsen et al., 2016). Generally, advantages are associated with being born closer to, but following, the organizational cut-off date utilized by each respective system. While the underlying mechanism(s) is/are undetermined, it is generally accepted that it is multifactorial in nature with subtle differences in physical, psychological, and emotional maturity leading to inequities in selection and development opportunities (Musch and Grondin, 2001; Cobley et al., 2009). A variety of individual, task, and environmental constraints can also influence the magnitude of RAEs and the degree to which they promote or hinder development on an individual level (see Wattie et al., 2015 for further discussion).

In the realm of sport, athletes often compete for membership on elite or representative teams. In a system that uses December 31st as a cut-off to group athletes, an athlete born in January is approximately 10% older than an athlete born at the end of the selection year (e.g., December) during their 10th year of life, leading to potential differences in lived experience, which can be compounded by considerable variability in biological maturity from childhood through to adolescence (Musch and Grondin, 2001; Malina et al., 2004). This may result in coaches and talent development scouts confusing increased size and experience for talent, leading to a higher likelihood of selection and all of the related opportunities that follow for relatively older athletes (e.g., enhanced coaching, training, and competition; Helsen et al., 1998). In contrast, athletes born later in the selection year may experience deselection and ultimately disengage from sport if unable to overcome the disadvantages associated with being relatively younger (MacDonald and Baker, 2013).

Sport researchers have given considerable attention to RAEs and the literature is saturated with documented birthdate trends that suggest these phenomena are present in a variety of sport contexts and age groups, among both male and female athletes around the world (see Cobley et al., 2009 and Smith et al., 2018 for reviews of male and female samples, respectively). Reports of increased awareness among sport administrators and the general public have also surfaced, including discussion of RAEs in popular media. For instance, Gladwell (2008) highlighted an over-representation of Canadian junior ice hockey players born early in the selection year in the opening chapter of his book entitled *Outliers: The Story of Success*. However, this increased attention has not produced equivalent action in mitigating their effects (Wattie et al., 2015). A variety of interventions have been put forth including the use of smaller (Boucher and Halliwell, 1991) or rotating age bands (Barnsley et al., 1985), corrective adjustments to objective measures of performance (e.g., sprint time; Romann and Cobley, 2015), and age-ordered shirt numbering to provide real time information for talent scouts (Mann and van Ginneken, 2017). Yet, few have been implemented and evaluated on a large enough scale to have a meaningful impact. For example, Helsen et al. (2012) reported no change over a 10-year period (2000–2001 to 2010–2011) in professional soccer despite increased awareness and knowledge of RAEs in sport, highlighting the pervasiveness of the effect. The persistence of RAEs and associated lack of action could theoretically contribute to a reduction in the talent pool for future advancement in sport, if relatively younger athletes do not have the opportunity to develop their skills and advance to higher levels. These athletes may also be subject to negative sport experiences with the end result being withdrawal from participation (e.g., Lemez et al., 2014).

Given the widespread popularity of the sport of soccer around the world and the associated pressure to achieve success in international competition, it is not surprising that RAEs have been recognized as an obstacle to optimal athlete development in this sport context. In fact, soccer has been one of the most common contexts for documentation of inequitable birth trends (Cobley et al., 2009). The subjective nature of athlete selections (i.e., player-to-player comparisons carried out on the field of play by scouts and coaching staff; Baker et al., 2014) create opportunities for relative age bias to surface from the developmental level to elite teams competing on the international stage. In an attempt to address relative age-related concerns inherent in talent development and selection activities, the U.S. Soccer Federation announced in 2015 that it would be changing its birth-year registration cut-off date from August 1st to January 1st. This change was introduced to align the U.S. youth soccer calendar with international standards, and simultaneously provide clearer information on player birthdates to “lessen” RAEs (U.S. Soccer, 2017), suggesting an intended benefit to participants at all levels. However, the magnitude of this policy change led to considerable controversy, with members of the soccer community taking to social media and website blogs (e.g., Woitalla, 2015), as well as the U.S. Youth Soccer (2016) website to voice their opinions and general unhappiness with this decision. Thus, the purpose of this study was to provide a summary of the online response from stakeholders^1^ (i.e., initial reactions and discussion) and the reported preliminary outcomes of this policy change at various levels of the sport, with attention to the manner in which the U.S. Soccer Federation framed (i.e., the underlying rationale for the decision) and publicly communicated (i.e., choice of mediums and language) its decision to change the annual cut-off date. Given the rarity of this type of change within a national sport organization and the extent of the impact at all levels of the sport (i.e., developmental to elite), the information gained from this analysis can inform future decisions of this nature at local, regional, and national governing levels across a variety of sport contexts.

## Materials and Methods

### Data Sampling and Collection

Data collection occurred from April to August 2017 (i.e., approximately 2 years after the announcement was made in 2015 and immediately prior to the deadline for implementation). Qualitative data (i.e., stakeholder posts in online forums) were collected manually from various social media platforms and website blogs^2^ (e.g., *Soccer America, World Class Coaching*). These sites were identified by searching “U.S. Youth Soccer” or “U.S. Soccer” in combination with specific key terms and phrases, including “cut-off date change,” “age group change,” “relative age effect,” “RAE,” “birthday cut-off,” and/or “rule change birth year.” When a potentially relevant online platform was identified (websites, *n* = 43; forums, *n* = 16; blogs, *n* = 4), the content was copied and pasted in its entirety into a master file. The compiled text (3,851 pages) was then reviewed in full to manually identify specific quotes related to any one of four criteria: (1) RAEs; (2) the associated policy change/announcement; (3) age group changes/issues, or; (4) organizational change. For example, if an online post stated that a player would gain/lose a developmental advantage as a result of the cut-off date change, the quote was retained for further analysis. Care was taken during this process to remove stakeholders' personal information from the compiled data (e.g., screen name, club name).

### Data Analysis and Re-presentation

The procedural steps of *hierarchical content analysis*, as outlined by Sparkes and Smith (2014) were followed to analyze the data collected from 63 social media sites. Content analysis is useful for investigations of relevance to practitioners and policy makers when the goal is to provide a comprehensive summary of phenomena, while staying close to the “surface” of the data (Sandelowski, 2000). From the 3,851 pages of text that were derived from online mediums, 262 stakeholder posts were manually identified based on their relevance to the aforementioned topic. A preliminary review was conducted to achieve familiarity with the compiled data (i.e., *immersion*); this included reading through each stakeholder post. *Raw data themes* were identified and labeled with *tags*. These tags were *clustered* to generate a list of higher order *sub-themes* and *categories*; this list was then applied to the data, *cross-checked*^3^ and reflexively modified to accommodate new insights when required to ensure the best fit for the data (Sparkes and Smith, 2014). Each respective tag (i.e., raw data theme) was assigned a maximum of one time per stakeholder post, but each post could contain one or more themes. A total of 404 unique passages of text were identified within the 262 stakeholder posts and categorized accordingly. Themes, sub-themes, and categories were organized in a table based on their hierarchical nature (as recommended by Sparkes and Smith, 2014), while being mindful of heterogeneity between each category. The themes, sub-themes, and categories were then summarized using descriptive statistics.

### Trustworthiness and Quality

The online posts were collected from a variety of social media platforms and website blogs to ensure representation from a variety of stakeholders. Four of the five authors were familiar with relative age research in the context of sport and were therefore able to identify valid themes and sub-themes in the raw data for subsequent analysis. However, the caveat of this familiarity was also recognized, that being the potential for personal bias in the construction of themes and the subsequent hierarchical organization of those themes. To address this limitation, a random selection of the compiled quotes (approximately one third of the total number of quotes) were independently evaluated by two authors^4^ and compared to assess the objectivity of the coding process. Disagreements were resolved through discussion and a revision of the coding structure ensued. A second round of comparison and *confirmation* (Sparkes and Smith, 2014) followed with another co-author^5^; this included critical dialogue and feedback on the compiled themes, sub-themes, and categories. A final review of the data followed to ensure consistent assignment of categories within the dataset.

## Results

Our qualitative content analysis revealed four main categories in the data collected from online discussion forums and blogs regarding the organizational policy change. These categories included stakeholder discussion, outcomes identified by stakeholders, recommended courses of action, and communication regarding the policy change (summarized in Table 1). *Stakeholder discussion* was the most common category identified with a total of 168 posts, which included 35 unique themes. These posts included general opinions and views regarding the impact of the policy change and related variables, but excluded any specific outcomes or recommendations. The most common higher order sub-theme, *interacting variables*, included discussion of moderating factors or systems that could, would, or already had an influence on the outcome(s) of the policy change. The *impact of social network* was frequently cited within this sub-theme and content focused on school-based cohorts (total of 13 posts) that would be disrupted as a result of the policy change. For example, one parent shared,

They were playing travel soccer with friends in their same grade from town and having a great time. Now knowing they will be on a mixed grade team, that their old team is being broken up and they will have to start all over….^6^

**Table 1:** Outline of study categories.

**Category**	**Definition**	**Total number of posts**
Stakeholder discussion	General opinions/views regarding the impact of the policy change and related variables	168
Outcomes identified by stakeholders	Explicit benefits or consequences of the policy change: Anticipated or presently experienced	148
Recommended courses of action	Recommendations provided by stakeholders: RAE-specific or related to the policy/organizational change	59
Communication regarding the policy change	Announcements or statements related to the unveiling/implementation of the policy change	29

Additional factors/systems that were perceived to be influential included club size (e.g., implementation could be more difficult for a smaller club), grade level (e.g., “senior” year), stage of development/chronological age, cut-off dates for the education system, college recruiting practices, and state-to-state variation (e.g., availability of soccer clubs).

Other commonly identified categories falling under the category of stakeholder discussion included a recognition that the bias associated with RAEs would persist and shift to a new group of athletes. For example, one stakeholder posted that, “U.S. Soccer has not made a more level playing field for players, they just shifted who is disadvantaged.” Further, it was recognized by some stakeholders that the mandate would have minimal impact with respect to talent development and identification activities. One stakeholder supported discussion with respect to a lack of impact specifically for reducing RAEs in the following post:

The idea that organizers are unaware of this effect does not seem to be a compelling reason for such a drastic change, as the effect has been well-documented and there are very few, if any, club directors I have met who are unaware of this.

A *perceived lack of consultation or concern* exhibited by the U.S. Soccer Federation during the decision-making process for the developmental levels vs. the elite was frequently expressed, as exemplified in the following example: “It is unfortunate that US Soccer has addressed the needs of a few hundred members (at most) and not the millions that I believe they should [be] supporting.” The lack of concern for stakeholder input (from any and all levels) also fell under this sub-theme. For instance, an unidentified representative at the state level posted, “It was difficult for us at the state association. We were not included in any conversations about this. We've tried to focus on how can we mitigate and make sure as few negative outcomes occur as possible.” Please refer to Table 2 for additional categories identified under stakeholder discussion.

**Table 2:** Category: stakeholder discussion.

**Theme**	**Number of posts**	**Higher order sub-theme**	**Number of posts**
Impact of club size^a^	3	Interacting variables	47
Impact of chronological age/developmental stage	6		
Impact of school cut-off dates^b^	7		
Impact of grade level^c^	9		
Impact of collegiate system/recruiting	7		
Impact of state-to-state variation	2		
Impact of social network^d^	13		
Minimal impact of the change (i.e., for talent identification)	6	Invalid rationale for policy change	25
Change not required	5		
Persistence of RAEs due to shift in bias	14		
Strength of current system	6	Characteristics of the current system on athlete development	15
Limitation of current system	9		
International competition	5	Levels of competition	19
Talent development teams	4		
High school vs. club soccer	6		
High school vs. elite/international soccer	1		
Competitive vs. recreational soccer	1		
Tournaments	2		
Developmental levels vs. elite	13	Lack of consultation or concern	25
Developmental levels vs. sport administration	1		
National level vs. all sport stakeholders	3		
National team vs. professional soccer	1		
Stakeholder input	5		
Proper athlete development	2		
Unanswered question^e^	3	Miscellaneous	37
Explanation of age groupings	3		
Explanation of USSF decision related to RAEs^f^	2		
Change is unfair	1		
Arbitrary plan/cut-off(s)	3		
Sport as a business	6		
Participation motive^g^	8		
Long term plan	1		
Clubs need to adjust quickly	1		
Situation overblown	2		
Undetermined meaning	7		

a *Includes specific discussion of impact on large vs. small clubs; emphasis on the discussion aspect vs. explicit outcomes of the policy change*.

b *Includes specific reference to a cut-off date*.

c *Typically associated with the negative stakeholder expectation – loss of season, team, or opportunity; in particular, the transition to high school*.

d *Predominantly classmates, but also community*.

e *Specific questions about the policy change*.

f *From stakeholder perspective*.

g *Includes specific reasons cited that children and/or youth choose to participate in sport; sometimes highlighted in contrast with the desire to become an elite athlete*.

*Outcomes identified by stakeholders* was the second most common category and included *explicit* benefits or consequences of the policy change, whether they were anticipated or actually (i.e., currently) being experienced by the stakeholder(s). Forty-six unique themes were identified within 148 stakeholder posts. The final tally from the data highlights that the actual *experienced impact* and *stakeholders' expectations* from implementation of the policy change were predominantly negative, outnumbering the positive outcomes 30:1 and ~2:1, respectively. An expectation of the *loss of a season, team, or opportunity* was most common (total of 18 posts) and predominantly associated with the transition to high school.

*Sport withdrawal* or *dropout* was also expected (14 individual posts) as an outcome of the policy change, whether it be from sport in general or soccer specifically through transfer to a new sport. A few stakeholders also recognized the adverse impact of dropout on other aspects of the sport (e.g., loss of revenue, decreased talent pool for future advancement to higher levels). For instance, one stakeholder identified the potential impact of the policy change on future involvement in soccer:

When they change the age brackets, how many kids stop playing? Now those kids may not grow up to be Landon Donovan, but they could grow up to be consumers of the game which drives revenue and sponsorship, they could grow up to be teachers of the game, facilitating the continued growth of the game by indoctrinating a new generation of fans. They could grow up to be board members of clubs that will shape the game for many others….

With respect to positive outcomes, there was a perceived benefit for *athlete development* mentioned by stakeholders (total of 11 posts). However, this benefit was sometimes tied to the athletes who would be relatively older (i.e., those with birthdates in January, February, and March). There was also a recognition that this mandate would benefit the national team by aligning with international competition structures. One stakeholder commented,

It also puts our players on the same age-playing calendar as the rest of the world so they will be used to competing in the right age-group. That makes it much easier for us to scout for the national teams and find players ready to compete internationally.

Other themes identified under this category can be found in Table 3.

**Table 3:** Category: outcomes identified by stakeholders.

**Theme**	**Number of posts**	**Higher order sub-theme**	**Number of posts**
Strengthen national team	6	Stakeholder expectations (positive)	38
Align internationally	5		
New opportunity	5		
Athlete development	11		
Decrease risk of injury	1		
Redistribute talent to enhance competition	2		
Elimination of age disadvantage^a^	2		
Provide information on RAEs/mitigate RAEs	3		
Athlete retention	2		
Independent teams^b^	1		
Cheating	1	Stakeholder expectations (negative)	77
Sport withdrawal/dropout^c^	14		
Increase RAEs	2		
Reduce access to sport	2		
Cannot play with friends/classmates	9		
Will not know teammates	1		
Detrimental to athlete development	1		
Change of team or club	6		
Athlete well-being	2		
Loss of season, team, or opportunity^d^	18		
Impact on small clubs	3		
New disadvantage	2		
Organizational chaos	2		
Confusion about age groupings	2		
Fear of playing with older or physically larger athletes	1		
Disruption to an existing team	5		
Coaches afraid to lose players^e^	1		
Impact on college recruiting/collegiate system	5		
Will not affect the growth of soccer	1	Stakeholder expectations (neutral)	1
New opportunity	1	Experienced impact (positive)	1
Teams folding/folded	2	Experienced impact (negative)	30
Small clubs could not realign teams in time	1		
Disruption to existing team	2		
Voting to fold club	1		
Unbalanced age groups	3		
Coach quit	1		
Adverse impact on coach(es)	2		
Cannot play with friends	1		
New disadvantage	1		
Athlete refusal to participate/attend practice	2		
Negative emotions (e.g., frustration, anger)	4		
Confusion about age groupings	5		
Confusion about timeline for implementation	1		
Sport withdrawal/dropout or reduced enrollment	3		
Fear of playing with older or physically larger athletes	1		
Change of club to one with more institutional support	1	Experienced impact (neutral)	1

a *Not specific to RAEs*.

b *With a focus on participation rather than elitism*.

c *Athletes and/or other stakeholders*.

d *Often related to the club vs. high school transition*.

e *Reason not specified*.

*Recommended courses of action* included recommendations from stakeholders which were generally directed to the U.S. Soccer Federation, but also toward other stakeholders (e.g., refusal to comply with the mandate). Some recommendations were RAE-specific, while others were related to the policy change, or organizational change in general (total of 26 unique themes in 59 stakeholder posts). Suggestions pertaining to the actual implementation of the policy change were most common with *grandparenting* being the preferred strategy (total of 11 stakeholder posts), meaning that the policy change should be implemented at the initiation levels of the sport (i.e., ages 4–6) and the cut-off date for older age groups remain unchanged. Stakeholders also recommended a longer transition time for implementation of the change, flexibility in the process/rule structure, and increased transparency at the national level.

A variety of strategies for creating athlete cohorts (i.e., *grouping strategies* such as half year age brackets and structuring by skill level) and promoting productive avenues for *organizational change* at the national level and beyond were also identified. For example, one stakeholder shared,

I think if we worried less about checking birth certificates and focused on structuring the sporting organizations around skill levels you would find that participation would increase and would last longer particularly for those at the lower end of the skill scale and at the same time continue to provide the challenges for those at the other end of the spectrum.

Further, the stakeholder data supported a need for increased access to all levels of the sport for all socioeconomic groups. Please refer to Table 4 for a detailed outline of themes under the category of recommended courses of action.

**Table 4:** Category: recommended courses of action.

**Theme**	**Number of posts**	**Higher order sub-theme**	**Number of posts**
Longer transition time	2	Implementation	23
Flexible process	2		
Flexible rules or structure for different levels of competition	6		
Grandparenting	11		
Transparency about policy change	1		
Communicate plan of action	1		
Remove age categories	3	Grouping strategies	14
Half year age brackets	2		
Multiple age groupings	1		
Structure by skill level	4		
Remove skill categories	1		
“Loose”/flexible cut-off	2		
Undetermined meaning	1		
Focus on athlete development	3	Organizational change	17
Athlete retainment	2		
Emphasize social aspect vs. talent development	2		
Emphasize enjoyment vs. winning	1		
Educate coaches on talent selection/identification	2		
Increase access to sport for all socioeconomic groups	3		
Serve all levels of participation	1		
Improve communication/coordination	2		
Transparency at the national level	1		
Refusal to comply	1	Other	5
Change age group labels	1		
Consider best solution based on club characteristics	1		
Undetermined meaning	2		

The final category of *communication regarding the policy change* included announcements or statements related to the unveiling of the policy change that were general in nature, but also included stakeholder posts related to any form of communication surrounding the policy change (refer to Table 5). Nine unique themes were identified within 29 stakeholder posts. A perceived *lack of communication* from the national level was noted in the data (total of 5 posts). For example, one stakeholder stated that, “The mandate was handed down with no parent input, and issued in a dictatorial fashion that unfortunately is a defining characteristic of U.S. Soccer.” Similarly, another individual commented,

They have screwed up this mandate from day one, passed it in secret 8 months before they said they would, have refused to even talk to the member organizations about it and have offered zero advice in how to smoothly implement them.

**Table 5:** Category: communication regarding the policy change.

**Theme**	**Number of posts**	**Higher order sub-theme**	**Number of posts**
Club/developmental level	6	General communication	13
National level	3		
Unknown/undetermined	4		
Club/development level	2	Communication issue	10
To the parents	1		
Lack of communication at the national level	5		
Misinformation from the national level	2		
Communicating for the purpose of effective planning^a^	2	Implementation process	6
General timing of changes	4		

a *Club level*.

Notably, two stakeholders shared club-level strategies utilized during the implementation process that could inform future policy changes of this nature. These quotes were thus coded as *communicating for the purpose of effective planning*. For instance, one commented,

I can't imagine a club more prepared. They have had multiple age group parent meetings to explain the process, play dates for age group players to further assess the players and build comfort among players that might not be new faces but don't know each other well. The staff has been evaluating the players all year as well in practice and games.

Likewise, another stakeholder posted,

At my club we got out in front of this. Met with all of our parents and held training sessions based on the birth year and the feedback we've been getting is that clearly communicating the changes, having a plan of action and getting out in front of it have aided in insuring our parents and players are less anxious about it.

Additional communication associated with the developmental level was general in nature and typically provided information regarding club policies or the timing of the implementation process.

## Discussion

### Overall Findings

The purpose of this study was to provide a summary of online stakeholder responses (i.e., initial reactions and discussion) to the U.S. Soccer Federation's decision to change the age group cut-off date from August 1st to January 1st, as voiced through social media and website blogs. The immediate impact was assessed to the extent possible through online mediums, and attention was given to stakeholders' perceptions of the manner in which the decision was framed and publicly communicated. Four categories emerged from the data, which included stakeholder discussion, outcomes identified by stakeholders, recommended courses of action, and communication regarding the policy change. In general, the actions of the U.S. Soccer Federation and related outcomes were negatively perceived by stakeholders.

The *impact of social network* developed through the education system was frequently cited and indeed, school-related friendships have been found to have a positive influence on athlete engagement (e.g., Fraser-Thomas et al., 2008). Friends were cited as the most common participation motive in the current study, followed by fun/enjoyment of the game. An emphasis on these aspects of participation is recommended during the early years of participation (e.g., 12 years of age and younger) and is often contrasted with a focus on elitism at young ages (see Côté and Abernethy, 2012 for further discussion). Thus, it is possible that the U.S. Soccer Federation may have contributed to a reduction in participation through an increased rate of dropout as a result of this policy change. The potential for this to occur was supported by qualitative data collected in this analysis (i.e., dropout categories were identified under the sub-themes of *experienced impact* and *stakeholder expectations*). A detailed participation analysis is not currently available in the published literature and this assertion is currently speculative. A decline in U.S. soccer participation coinciding with implementation of the policy change has been reported by various sources (e.g., Sports Fitness Industry Association, 2018; Lange, 2020), but needs to be confirmed using longitudinal data. Further, a detailed examination of the underlying contributors to this trend may be informative for the U.S. Soccer Federation and other national level sport governing bodies who may be contemplating a similar policy change, as increased dropout rates could theoretically affect the talent pool available for future advancement to international levels of competition.

“*Grandparenting,”* whereby changes are implemented gradually beginning in the initiation years of participation (i.e., ages 4–6), may have been a feasible solution for consideration and was recommended by multiple stakeholders. However, the data suggest this option was not made available to local clubs and opportunities for stakeholder input were limited. Further, a perceived lack of “transparency” and *lack of concern/consultation* for the developmental levels (vs. the elite) surfaced in the content of stakeholder posts; this occurred despite the recognition by some stakeholders that the change would indeed provide benefits at the highest levels of international competition.

### Relative Age Considerations

With respect to RAEs, it was recognized by several stakeholders that the policy change would not resolve the inequitable selections and provision of opportunities afforded to those who are relatively older (e.g., Cobley et al., 2009), and was thus not an appropriate rationale for this policy change. The assertion that relative age bias would simply shift to a new group of athletes is supported by previous research. Helsen et al. (2000) analyzed birthdate distributions for national youth league players between the ages of 10 and 18 years, after the Belgian Soccer Federation implemented a similar cut-off date change. Among 10 to 16-year-olds, the athletes born in the early part of the *new* selection year (i.e., those born in January through March) were more likely to be identified as talented compared to those born later (i.e., August through October) and who had previously been advantaged only 1 year earlier. The researchers attributed this shift in selection advantages to athletes' physical size rather than differences in their skill or talent levels, as supported by a previous examination of Belgian youth soccer players (Helsen et al., 1998).

Several of the recommendations proposed by stakeholders were aligned with relative age literature, showing an awareness of RAEs. However, there continues to be a lack of appropriate, interventive action at the developmental levels of sport (Wattie et al., 2015; Mann and van Ginneken, 2017). For instance, smaller age brackets (Boucher and Halliwell, 1991) and removal of skill categories prior to the completion of maturation (Baker et al., 2010) have been proposed in previous work. This observation also indirectly supports the notion that increasing general awareness of RAEs is not enough to mitigate their impact (see Helsen et al., 2012 for further discussion).

### Recommendations for Future Policy Changes

While a change to an annual age-group cut-off date is a rare event, the findings of this analysis can inform other types of organizational change in sport at various levels of participation (e.g., from the grassroots level to talent development academies). Further, the findings of this study add to the limited body of research examining the implementation of organizational change at the youth sport level and provide support for recommendations in previous literature.

Legg et al. (2016) examined the impact of a province-wide change initiated by *Ontario Soccer* (previously known as the *Ontario Soccer Association*), aimed at improving *Long-Term Player Development* in Ontario, Canada. A case study approach was utilized with study participants recruited from one of four groups: staff members from Ontario Soccer, board members at two local-level soccer clubs, coaches, and parents. Factors that both aided and constrained the change were identified; and similarities to the present analysis exist. First, the importance of formal communication from the sport governing body was highlighted. Qualitative data in this study suggest communication from the U.S. Soccer Federation was limited. However, this organization should have been the “dominant voice” in the process to both educate and communicate the new procedures to be followed (Danylchuk et al., 2015; Legg et al., 2016). Misinformation from the national level was also reported. Specifically, unreliable information was cited with respect to cut-off dates in other countries, along with errors in the new age divisions outlined prior to implementation. The importance of communication is also supported by anecdotal reports from representatives in two unnamed clubs in this study; specifically, holding meetings with parents and communicating a plan of action were perceived to be effective in easing concerns about the policy change. However, a lack of information at the club/developmental level was also reported, suggesting accurate, consistent (i.e., from all levels of the sport), and timely information was needed for all stakeholders (e.g., athletes, parents, coaches) to facilitate implementation.

The second recommendation that can be supported based on the current data is the endorsement of stakeholder involvement from all levels in the decision-making process. For example, suggestions for grandparenting or a “phase-in approach” were made and may have been met with a greater degree of acceptance of the mandate by stakeholders at developmental levels (Legg et al., 2016). McVea and Freeman (2015) argued for the importance of stakeholder “relationships” (as opposed to inferior “roles”) in creating value during organizational decision-making, suggesting stakeholders have an important role to play in the success of any organization in benefitting those affected by organizational activities. When conflicts arise (as they often do), organizational leaders must adapt to create as much value as possible for these stakeholders (Freeman et al., 2010). Acknowledging that an examination of U.S. Soccer's specific efforts to gain stakeholder input was beyond the scope of this study, the perceived *lack of consultation or concern* for the developmental levels inherent in the current data may be concerning. Stakeholders should be consulted and involved in strategic planning, actively engaged in achieving outcomes, and well-informed regarding organizational activities (e.g., Australian Sports Commission, 2012).

### Strengths and Limitations

Obtaining the current data from social media and website blogs provided a novel collection method that may avoid or minimize the responder bias that might be present in a more traditional research setting. Further, social media and other modes of internet communication provide more options for sharing opinions (Picard, 2015), permitting the sampling of a widespread and diverse population (i.e., the U.S. Soccer community across various levels of participation and different regions of the country). However, it is acknowledged that this analysis is limited to stakeholders who chose to express their opinions in online forums and thus, may not have been fully representative of all stakeholders impacted by the change (e.g., the youngest U.S. soccer participants). These online data may also be limited by the fluidity of stakeholder opinions. For example, some posts had a negative emotional tone and may have been written in the “heat of the moment.” Ideally, follow-up assessments would be conducted after a period of time had passed. This was not feasible in the design of the current study. Furthermore, elements of social media (e.g., anonymity) tend to lend themselves to “belligerent venting of anger” (Picard, 2015, p. 38) and individuals tend to be drawn to negative content (Acerbi, 2019). This may account for the high number of negative views/opinions (vs. positive content) that was noted in the collected data.

The use of qualitative (hierarchical) content analysis provided a comprehensive, descriptive summary of the data that can inform future policy change. However, this method of analysis was limited in this study because in most cases, it was not possible to identify *who* made the post in the online forum. For instance, it would be informative to know whether the stakeholder was an athlete, parent, coach, or administrator, along with relevant demographic information (e.g., age, gender, level of competition, degree/length of involvement in the sport, volunteer or paid coach/administrator). This information could provide insight into how organizational change(s) could be tailored at various levels of participation for greater effectiveness and ease of implementation.

Additional limitations associated with content analysis include the possibility for tagging of raw data themes to separate an important section of text from the context in which it was delivered by the stakeholder (Sparkes and Smith, 2014). Care was taken during the coding process to minimize this by isolating distinct sections within a stakeholder post in proximity to the rest of the passage (i.e., stakeholder posts were divided into multiple sections, and labeled a, b, c, as required). Homogeneity within themes and sub-themes were carefully reviewed following this process. The descriptive statistics utilized may give the impression that “more is better” (Sparkes and Smith, 2014) and it is acknowledged that this may or may not be the case. Highly relevant themes emerged in smaller numbers in this study (e.g., communication at the club level) and these were discussed accordingly in the paper. Along with the aforementioned limitations, this study is limited by the researcher bias that can occur in any type of qualitative analysis. Attempts were made to minimize this potential source of bias by conducting two rounds of comparison (i.e., between the first and second/third authors). This process included critical dialogue between the authors.

### Future Directions

In this study, the content of the qualitative data suggested that the majority of stakeholders had recently become aware of the policy change at the time of posting on the social media/website blog or were currently in the initial stages of implementation. Thus, post-implementation outcomes and follow-up would be valuable. Further qualitative analysis at various levels of U.S. soccer would be informative. For instance, interviews with U.S. Soccer Federation representatives might enhance understanding of the contributing factors influencing the decision process for this mandate. Case studies with athletes, parents, coaches, and administrators would be helpful and could provide additional evidence to support or refute the findings of this study. This could also include analysis of formal press releases from member organizations and/or club-level policy changes occurring subsequent to the U.S. Soccer mandate. Quantitative analyses of dropout subsequent to implementation of the policy change, along with examination of associated factors (e.g., loss of revenue, reduced volunteerism) would be informative for sport governing bodies including the U.S. Soccer Federation, but also for local and regional level sport organizations due to the potential impact of sport withdrawal at all levels of participation.

### Conclusions

This study examined the online stakeholder response to the U.S. Soccer Federation's decision to change the organizational cut-off date to align with international standards and “lessen” RAEs in the sport. Stakeholder reactions were generally negative, and resistance to the change may have been reduced through better communication from the national level and opportunities for stakeholder involvement. Further, post-implementation analyses would be valuable using a variety of methodological approaches.

## Data Availability Statement

The raw data supporting the conclusions of this article will be made available by the authors, without undue reservation.

## Author Contributions

JD, SH, and LC designed the study. SS collected and cleaned the data. KS coded the data (with assistance from LC and SS), summarized the results, and drafted the manuscript. All authors participated in the editing process and have approved the final version of the manuscript.

## Conflict of Interest

The authors declare that the research was conducted in the absence of any commercial or financial relationships that could be construed as a potential conflict of interest.

## Footnotes

^1^In this study, the term *stakeholder* refers to any individual who expressed an opinion in an online forum regarding the U.S. Soccer Federation's policy change and may include (but is not limited to), athletes/soccer participants, parents, grandparents, coaches, board members in local/community soccer clubs, and representatives from sport governing agencies at various levels (e.g., state, regional, national). All stakeholders identified during data collection were given equal weight in this study.

^2^The data collected were publicly available and therefore, research ethics clearance was not required to conduct this study.

^3^The cross-checking process, whereby the raw data themes and clusters of sub-themes are thoroughly examined (Sparkes and Smith, 2014), was initially conducted by the first author, with additional rounds of comparison and eventual confirmation performed by the second and third authors (outlined further in the Trustworthiness and Quality section).

^4^Independent coding of the data was conducted by the lead author (familiar with relative age research) and the first round of comparison was completed with the second author (familiar with the data but not relative age research, *per se*).

^5^The second round of comparison was conducted in collaboration with the third author (familiar with relative age research).

^6^The latter portion of this quote was coded as a negative outcome (i.e., disruption to existing team) and was included to maintain the context of the stakeholder post.
